# Statins and clinical outcomes in hospitalized COVID-19 patients with and without Diabetes Mellitus: a retrospective cohort study with propensity score matching

**DOI:** 10.1186/s12933-021-01336-0

**Published:** 2021-07-10

**Authors:** Prateek Lohia, Shweta Kapur, Sindhuri Benjaram, Zachary Cantor, Navid Mahabadi, Tanveer Mir, M. Safwan Badr

**Affiliations:** Department of Internal Medicine, Wayne State University, Detroit, MI 48201 USA

**Keywords:** Statins, COVID-19, Mortality, Diabetes Mellitus, Inpatient, Mechanical ventilation, Intensive care, Race

## Abstract

**Background:**

The pleiotropic effects of statins may reduce the severity of COVID-19 disease. This study aims to determine the association between inpatient statin use and severe disease outcomes among hospitalized COVID-19 patients, especially those with Diabetes Mellitus (DM).

**Research design and methods:**

A retrospective cohort study on hospitalized patients with confirmed COVID-19 diagnosis. The primary outcome was mortality during hospitalization. Patients were classified into statin and non-statin groups based on the administration of statins during hospitalization. Analysis included multivariable regression analysis adjusting for confounders and propensity score matching to achieve a 1:1 balanced cohort. Subgroup analyses based on presence of DM were conducted.

**Results:**

In the cohort of 922 patients, 413 had a history of DM. About 27.1% patients (n = 250) in the total cohort (TC) and 32.9% patients (n = 136) in DM cohort received inpatient statins. Atorvastatin (n = 205, 82%) was the most commonly prescribed statin medication in TC. On multivariable analysis in TC, inpatient statin group had reduced mortality compared to the non-statin group (OR, 0.61; 95% CI, 0.42–0.90; p = 0.01). DM modified this association between inpatient statins and mortality. Patients with DM who received inpatient statins had reduced mortality (OR, 0.35; 95% CI, 0.21–0.61; p < 0.001). However, no such association was noted among patients without DM (OR, 1.21; 95% CI, 0.67–2.17; p = 0.52). These results were further validated using propensity score matching.

**Conclusions:**

Inpatient statin use was associated with significant reduction in mortality among COVID-19 patients especially those with DM. These findings support the pursuit of randomized clinical trials and inpatient statin use appears safe among COVID-19 patients.

**Supplementary Information:**

The online version contains supplementary material available at 10.1186/s12933-021-01336-0.

## Introduction

The global pandemic of coronavirus disease 2019 (COVID-19), caused by severe acute respiratory syndrome coronavirus-2 (SARS-CoV-2), continues to take a toll on human lives all around the world. Several risk factors including old age, Diabetes Mellitus (DM), obesity, metabolic syndrome, and obstructive sleep apnea have been associated with poor outcomes in COVID-19 [1–5]. Worse COVID-19 outcomes have been reported in Latino and Black populations highlighting the socioeconomic and racial disparities in health care [6–8]. DM is a prevalent chronic condition affecting close to 10.5% of the United States (US) population and its prevalence has been steadily increasing for the past few decades [9]. Black adults are 60% more likely to be diagnosed with DM than non-Hispanic white adults [10].

Statins are usually the first line of treatment as lipid-lowering agents and are used widely in patients with cardiovascular risks. Apart from their lipid-lowering effects, statins have also been known for their immunomodulatory, anti-inflammatory effects, and vasculo-protective effects [11–17], and these pleiotropic effects of statins have prompted exploration of any possible benefits of statins in the COVID-19 disease. An early study conducted in China noted that inpatient statin use was associated with decreased mortality in COVID-19 patients [18]. Subsequently, multiple retrospective cohort studies, including one conducted by us, noted the association between antecedent statin use and decreased mortality in COVID-19 patients [19–23]. However, none of these studies looked at the inpatient statin therapy and continuation of statins in the inpatient setting among hospitalized COVID-19 patients.

However, literature is scarce on the role of statins in patients with DM developing COVID-19 infection. The study by Saeed et al. [24] noted that inpatient statin use was associated with decreased mortality in patients with DM and COVID-19, whereas the results from CORONADO study [25] suggest increased mortality among the antecedent statin users in COVID-19 patients with DM. These conflicting results led us to explore the association of inpatient statin use with mortality, need for intensive care unit (ICU) admission, and mechanical ventilation in the patients hospitalized with COVID-19, among a majority Black patient population. We also explored if the association between statins and clinical outcomes was any different in the subset of patients with DM. Additionally, we also looked at the intensity-response relationship of inpatient statins in COVID-19 patients. We also investigated if the continuation of statin therapy in the inpatient setting has any association with clinical outcomes among the COVID-19 patients who were using antecedent statins as home medication.

## Research design and methods

### Study design

This retrospective cohort study was conducted on 922 adult patients hospitalized with COVID-19 diagnosis. This study was approved by the Detroit Medical Center (DMC) and Wayne State University institutional review board. No external funding was received for conducting the study.

### Study site and patient population

Adult patients (≥ 18 years of age) who presented to the hospital and had a laboratory-confirmed COVID-19 diagnosis (either via nasopharyngeal or oropharyngeal swab) were included in the study. The data were collected from COVID-19 positive patients who presented to the two DMC hospitals, located in the Detroit metropolitan area catering to an underserved, majority Black population.

### Data collection

**Fig. 1:**
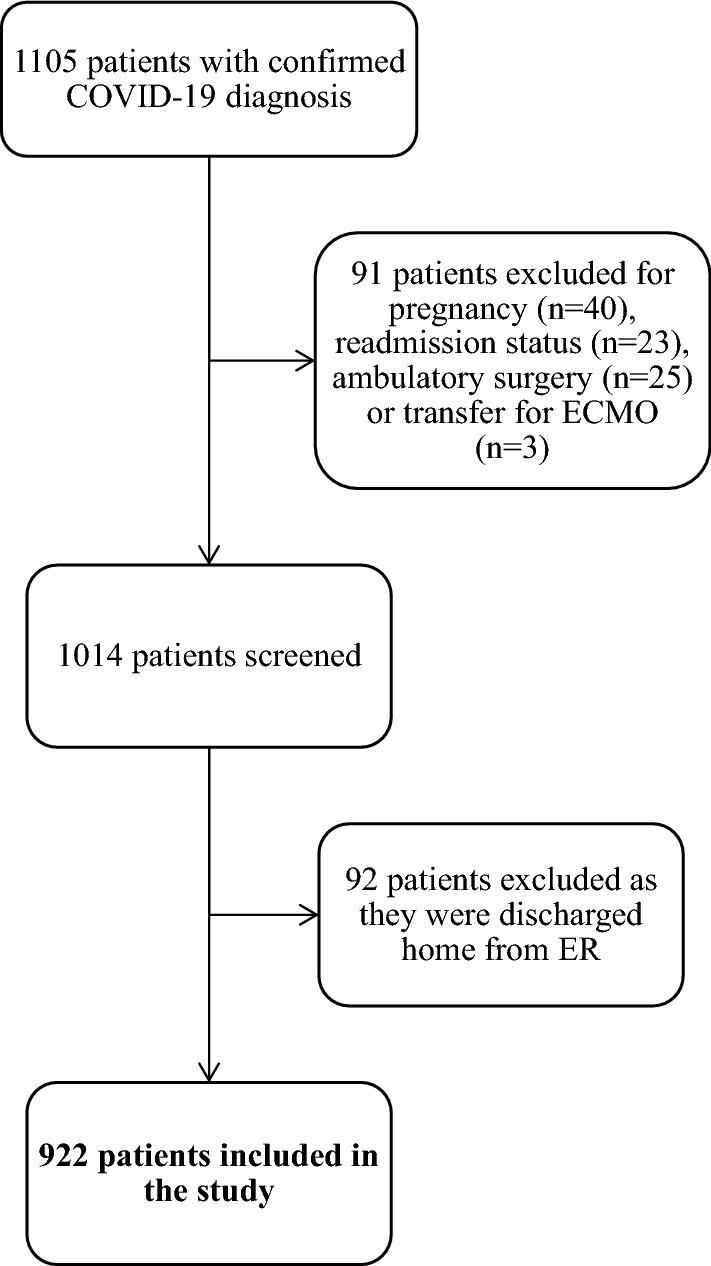
Flowchart depicting patient inclusion criteria. Adult patients (≥ 18 years of age) with a confirmed COVID-19 diagnosis were included. Patients under the age of 18, any readmission during the time frame, ambulatory surgery, pregnant patients and patients transferred to an outside facility for extracorporeal membrane oxygenation were excluded from the study. Additionally, patients discharged from the Emergency department were also excluded

A list of 1105 consecutive adult patients (≥ 18 years of age) who were admitted to the hospital between March 10, 2020, and June 30, 2020, and had a laboratory-confirmed COVID-19 PCR diagnosis was collected in collaboration with the institutional information technology services. Any readmissions during the time frame, ambulatory surgery patients, and pregnant patients were excluded from the study. Patients transferred to an outside facility for extracorporeal membrane oxygenation (ECMO) therapy were also excluded. Additionally, patients who did not require inpatient admission and were discharged from the Emergency Department (ED) visit were excluded. After following the above exclusion criteria, a total of 922 patients were included in the study, as shown in Fig. 1.

Electronic medical records were thoroughly reviewed for all patients included in the study. Data points were manually collected and coded for each patient. To ensure data integrity and accuracy, randomly selected patient charts were additionally verified by the principal investigator. Demographic data collected included age, sex, race, and insurance status. Additionally, the BMI and smoking status were noted for each patient. The information was collected for 11 prominent comorbidities (as defined in Additional file 1: Table S1) including DM, hypertension, coronary artery disease (CAD), congestive heart failure (CHF), preexisting lung diseases, any history of cancer, chronic liver disease, chronic kidney disease (CKD), end-stage renal disease (ESRD) on dialysis, hyperlipidemia and history of prior stroke. The time from the onset of symptoms to the day of presentation to the hospital was also noted for each patient, when available. Based on this data, days from symptom onset to presentation were classified as less than 5, 6–10,11–15, and 16 or more days.

The exposure in this study was the use of inpatient statins among the hospitalized COVID-19 patients. The data on inpatient statin medication were obtained from the active medication lists during hospitalization. Based on whether the patient received statins during the inpatient stay or not, the patients were classified into two groups, statin group (patients who received statins during hospitalization) and non-statin group (patients who did not receive statins during their inpatient stay). The type and intensity of statins were noted for patients who received inpatient statins and they were classified into low, moderate, or high intensity statin based upon the American College of Cardiology and American Heart Association classification of statin intensity [26]. Information regarding the antecedent use of statins (i.e. history of the use of statin medications at home) was also collected from the list of home medications. For patients with documented use of statins as home medication, it was further noted whether statin therapy was continued in the hospital or not, along with the reason for discontinuation, when available. The nature and clinical course of the patient’s hospitalization were also noted. It was also noted if the patients received antibiotics, corticosteroids, remdesivir, and therapeutic anticoagulation during their hospitalization. Information about age, systolic and diastolic blood pressure, respiratory rate, Blood urea nitrogen (BUN), confusion at the time of admission to the hospital was used to calculate the CURB-65 score at presentation for each patient.

### Outcomes

The primary outcome for this study was mortality during hospitalization and the secondary outcomes were the need for mechanical ventilation, and ICU admission. All of the patients included in the study had a documented acute care endpoint (mortality/discharged status) at the time of data collection.

### Statistical analysis

Descriptive analysis was done to describe categorical variables as frequency and percentages, and continuous variables as median and interquartile range. Baseline characteristics of the two groups- statin and non-statin group, were compared using Mann–Whitney *U* test for continuous variables (with non-normal distribution) and Chi-square test for the nominal variables. A crude relative association measure (Odds ratio, OR) was calculated for each correlation using the Pearson chi-square test. An adjusted odds ratio was calculated using binary logistic regression. In the fully adjusted models, adjustments were made for age, sex, race, BMI, smoking status, insurance, CURB-65 score at presentation, days from symptom onset to presentation, and prior comorbidities including DM, hypertension, CAD, CHF, preexisting lung diseases, any history of cancer, chronic liver disease, CKD, ESRD, hyperlipidemia, and history of prior stroke. Age and BMI were taken as continuous variables, CURB-65 score as ordinal variable while the remaining were categorical variables. We also checked for the interaction between DM and inpatient statins. A p-value of less than 0.05 was determined to be significant. Stepwise regression using forward selection (Wald) method was also performed to obtain an optimal model and further validate the findings. The optimal model only included the variables that contributed significantly to the model.

To minimize any known confounding due to selection bias, propensity score matching was done. Propensity score matching was performed to obtain a 1:1 matched cohort using the ‘nearest-neighbor’ approach without replacement, with a match tolerance of 0.05. Propensity score was defined as the predicted probability of the patient receiving inpatient statins given the patient's demographics, BMI, severity at presentation, and comorbidities. The covariates used to calculate the propensity score included age, sex, Black race, BMI, insurance, smoking status, days from symptom onset to presentation to the hospital, CURB-65 score at presentation, DM, hypertension, CAD, CHF, preexisting lung diseases, any history of cancer, chronic liver disease, CKD, ESRD, hyperlipidemia, and history of prior stroke. Baseline characteristics of the two groups were compared for the matched cohort to validate the propensity score model. The aim was to achieve a balanced distribution of all the covariates in the propensity score matched (PSM) cohort. If any of the baseline characteristics failed to achieve the balanced distribution after PSM, then double robust approach was used to remove any residual confounding bias [27]. Binary logistic regression was conducted on the PSM cohort for all the outcomes.

Subgroup analyses were conducted for patients with DM and patients without DM, as the interaction between DM and inpatient statins was found to be significant in the multivariable regression model for the total cohort (p = 0.016). Descriptive analyses were conducted for both the subgroups. PSM was also performed on both the DM and the non-DM cohort using the approach similar to the one described above, and regression was conducted on the two PSM cohorts (PSM DM cohort and PSM non-DM cohort). Further analyses were performed on the subset of patients who were using statins as home medication at the time of presenting to the hospital (i.e. the patients with a history of antecedent statin use), to ascertain if the continuation of statin therapy in the inpatient setting had any association with the clinical outcomes of COVID-19 in these patients. None of the variables used in the statistical analyses were missing more than 5% of values. Hence, no imputations were done for the missing data. Statistical analyses were completed using IBM SPSS software version 27.

## Results

### Baseline characteristics and clinical course of the total cohort

The total cohort of this study consisted of 922 patients with Blacks (n = 688, 74.6%) being the predominant race. Median age of the total cohort was 66 years (IQR- 56–73.25 years). Median BMI was 29.4 kg/m^2^ (IQR-25.2–35.9) and the majority of patients were in the obese category (n = 432, 46.9%). Most common comorbidities in this cohort were hypertension (n = 756, 82%), DM (n = 413, 44.8%) and preexisting lung diseases (n = 304, 33%). More than 70% of the patients presented to the hospital within 5 days of symptom onset (n = 649, 70.45%), and 84.3% of the patients (n = 777) had a CURB-65 score of 2 or less at presentation. A total of 250 patients (27.1%) received inpatient statins during hospitalization and atorvastatin (n = 205, 82%) was the most commonly prescribed statin medication. The details of corticosteroids, remdesivir, antibiotics, and therapeutic anticoagulation received during hospitalization have been summarized in Additional file 1: Table S2. The distribution of the patients who received these treatments were similar in both the statin and non-statin group. Patients in the statin group were older and had a more severe burden of comorbidities such as DM, CAD, hypertension, hyperlipidemia, CHF, stroke and CKD. The details of the baseline characteristics of the total cohort have been summarized in Table 1. All these differences in the baseline characteristics were accounted for in the statistical analysis using both the traditional multivariable regression models as well as the propensity score matching. About 16.6% of the patients (n = 153) were admitted directly to ICU from the ER and an additional 182 patients were transferred to ICU from the inpatient floors. During the course of hospitalization, 31.8% of the patients (n = 293) died, 26.9% of the patients (n = 248) needed mechanical ventilation and 36.3% of the patients (n = 335) required ICU admission. The details of the clinical course of the total cohort have been summarized in Table 2. Details about the type and intensity of statins received during hospitalization are summarized in Additional file 1: Table S3.

**Table 1:** Baseline characteristic of patients

		Unmatched			Propensity score matched		
Characteristic	Cohort (n = 922)	Inpatient Statin (n = 250)	Non Statin (n = 672)	p-value	Inpatient Statin (n = 229)	Non Statin (n = 229)	p-value
Age, (years) n (%)							
Median (IQR)	66 (56–73.25)	66 (59–75)	65 (54–73)	0.007	66 (58.5–75)	68 (59–75)	0.49
18–30	26 (2.8)	0	26 (3.9)		0	5 (2.2)	
31–45	81 (8.8)	10 (4)	71 (10.6)		8 (3.5)	20 (8.7)	
46–64	320 (34.7)	91 (36.4)	229 (34.1)		87 (38)	63 (27.5)	
≥ 65	495 (53.7)	149 (59.6)	346 (51.5)		134 (58.5)	141 (61.6)	
Sex, n (%)							
Male	490 (53.1)	139 (55.6)	351 (52.2)	0.36	123 (53.7)	128 (55.9)	0.64
Female	432 (46.9)	111 (44.4)	321 (47.8)		106 (46.3)	101 (44.1)	
Race, n (%)							
Blacks	688 (74.6)	179 (71.6)	509 (75.7)	0.2	167 (72.9)	172 (75.1)	0.59
Other races	234 (25.4)	71 (28.4)	163 (24.3)		62 (27.1)	57 (24.9)	
BMI							
Median (IQR)	29.4 (25.2–35.9)	29.25 (24.4–34.83)	29.4 (25.6–36.5)	0.17	29.7 (24.9–35.1)	29.27 (25.65–35.35)	0.78
< 18.5 (underweight)	28 (3)	8 (3.2)	20 (3)		6 (2.6)	8 (3.5)	
18.5–24.9 (normal)	195 (21.1)	65 (26)	130 (19.3)		53 (23.1)	44 (19.2)	
25–29.9 (overweight)	266 (28.9)	61 (24.4)	205 (30.5)		57 (24.9)	73 (31.9)	
≥ 30 (obese)	432 (46.9)	116 (46.4)	316 (47)		113 (49.3)	104 (45.4)	
Comorbidities, n (%)							
Preexisting lung diseases	304 (33)	87 (34.8)	217 (32.3)	0.47	81 (35.4)	74 (32.3)	0.49
Coronary artery disease	214 (23.2)	94 (37.6)	120 (17.9)	< 0.001	86 (37.6)	76 (33.2)	0.33
Hypertension	756 (82)	233 (93.2)	523 (77.8)	< 0.001	213 (93)	200 (87.3)	0.04
Diabetes Mellitus	413 (44.8)	136 (54.4)	277 (41.2)	< 0.001	126 (55)	119 (52)	0.51
Hyperlipidemia	280 (30.4)	127 (50.8)	153 (22.8)	< 0.001	109 (47.6)	101 (44.1)	0.45
Congestive Heart Failure	135 (14.6)	56 (22.4)	79 (11.8)	< 0.001	51 (22.3)	46 (20.1)	0.57
Stroke	100 (10.8)	48 (19.2)	52 (7.7)	< 0.001	39 (17)	37 (16.2)	0.8
Cancer	88 (9.5)	18 (7.2)	70 (10.4)	0.14	18 (7.9)	16 (7)	0.72
Chronic liver disease	35 (3.8)	3 (1.2)	32 (4.8)	0.01	3 (1.3)	5 (2.2)	0.48
Chronic kidney disease	110 (11.9)	41 (16.4)	69 (10.3)	0.01	36 (15.7)	37 (16.2)	0.9
ESRD on dialysis	98 (10.6)	31 (12.4)	67 (10)	0.29	30 (13.1)	20 (8.8)	0.13
Smoking	387 (42)	117 (46.8)	270 (40.2)	0.07	103 (45)	112 (48.9)	0.4
Insurance							
Uninsured	9 (1)	3 (1.2)	6 (0.9)	0.03	3 (1.3)	4 (1.7)	0.83
Medicaid	242 (26.2)	52 (20.8)	190 (28.3)		50 (21.8)	45 (19.7)	
Medicare	595 (64.5)	180 (72)	415 (61.8)		161 (70.3)	168 (73.4)	
Private	76 (8.2)	15 (6)	61 (9.1)		15 (6.6)	12 (5.2)	
Days from symptom onset to hospital presentation							
0–5	649 (70.4)	185 (74)	464 (69)	0.2	173 (75.5)	168 (73.4)	0.78
6–10	160 (917.4)	39 (15.6)	121 (18)		39 (17)	39 (17)	
11–15	55 (6)	10 (4)	45 (6.7)		10 (4.4)	15 (6.6)	
16 +	18 (2)	7 (2.8)	11 (1.6)		7 (3.1)	7 (3.1)	
CURB-65 at presentation							
0	222 (24.1)	36 (14.4)	186 (27.7)	< 0.001	35 (15.3)	44 (19.2)	0.44
1	280 (30.4)	81 (32.4)	199 (29.6)		76 (33.2)	62 (27.1)	
2	275 (29.8)	88 (35.2)	187 (27.8)		82 (35.8)	80 (34.9)	
3	110 (11.9)	38 (15.2)	72 (10.7)		30 (13.1)	32 (14)	
4	20 (2.2)	4 (1.6)	16 (2.4)		3 (1.3)	8 (3.5)	
5	12 (1.3)	3 (1.2)	9 (1.3)		3 (1.3)	3 91.3)

**Table 2:** Clinical course of patients

Characteristic	Cohort	Unmatched		Propensity score matched	
		Statin	Non-Statin	Statin	Non-Statin
Total cohort					
Mortality	293 (31.8)	69 (27.6)	224 (33.3)	59 (25.8)	97 (42.4)
Mechanical ventilation	248 (26.9)	52 (20.8)	196 (29.2)	48 (21)	78 (34.1)
ICU admission	335 (36.3)	76 (30.4)	259 (38.5)	70 (30.6)	102 (44.5)
Diabetes mellitus patients					
Mortality	152 (36.8)	34 (25)	118 (42.6)	32 (24.8)	58 (45)
Mechanical ventilation	146 (35.4)	32 (23.5)	114 (41.2)	31 (24)	54 (41.9)
ICU admission	179 (43.3)	47 (34.6)	132 (47.7)	45 (34.9)	62 (48.1)
Non-diabetes mellitus patients					
Mortality	141 (27.7)	35 (30.7)	106 (26.8)	30 (31.9)	31 (33)
Mechanical ventilation	102 (20)	20 (17.5)	82 (20.8)	17 (18.1)	20 (21.3)
ICU admission	156 (30.6)	29 (25.4)	127 (32.2)	25 (26.6)	35 (37.2)

After propensity score matching in the total cohort, a 1:1 balanced cohort of 458 patients was obtained, out of which 229 patients received inpatient statins while 229 patients did not receive any inpatient statins. In the propensity score matched (PSM) cohort, the distribution of the baseline characteristics between the two groups was similar for all covariates, except hypertension (p = 0.04). About 93% of the patients (n = 213) in the statin group had documented history of hypertension compared to 87.3% of the patients in the non-statin group (n = 200).

### Inpatient statins and clinical outcomes in the total cohort

**Fig. 2:**
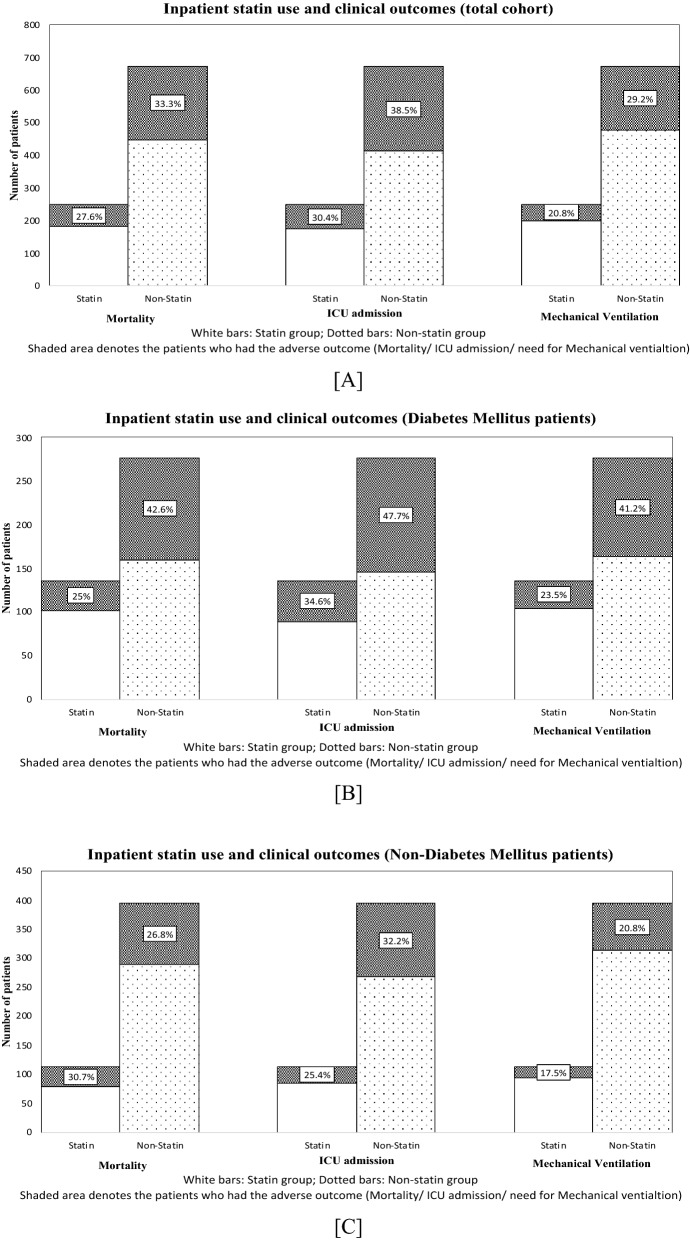
Bar graphs depicting clinical outcomes in the statin and non-statin groups of hospitalized COVID-19 patients, **A** represents Total cohort, **B** represents patients with Diabetes Mellitus and **C** represents patients without Diabetes Mellitus. Percentages shown in the shaded area denote the proportion of patients in that group who had the severe outcome (mortality/ ICU admission/need for mechanical ventilation)

In the unadjusted analysis, inpatient statin use had significant association with decreased need for ICU admission (OR, 0.70; 95% CI, 0.51–0.95; p = 0.02), and reduced need for mechanical ventilation (OR, 0.64; 95% CI, 0.45–0.90; p = 0.01) (Fig. 2A). However, the association between inpatient statin use and mortality was not statistically significant in the unadjusted model (OR, 0.76; 95% CI, 0.55–1.05; p = 0.1). In multivariable regression model, after adjusting for age, sex, race, BMI, insurance, days from symptom onset to presentation, CURB-65 score at presentation, smoking status, and comorbidities, inpatient statin use was associated with a significant reduction in mortality (OR, 0.61; 95% CI, 0.42–0.90; p = 0.01), reduced need for ICU admission (OR, 0.61; 95% CI, 0.43–0.87; p = 0.006), and a decreased need for mechanical ventilation (OR, 0.54; 95% CI, 0.36–0.80; p = 0.002). The results of stepwise regression further validate these findings using an optimal model and have been summarized in Table 3.

**Table 3:** Association between inpatient statins and severe disease outcomes- Mortality, Mechanical ventilation and ICU admission

Characteristic	Mortality		ICU Admission		Mechanical ventilation	
	OR (95% CI)	p-value	OR (95% CI)	p-value	OR (95% CI)	p-value
Total cohort						
Unadjusted	0.76 (0.55–1.05)	0.1	0.70 (0.51–0.95)	0.02	0.64 (0.45–0.90)	0.01
Fully adjusted*	0.61 (0.42–0.90)	0.01	0.61 (0.43–0.87)	0.006	0.54 (0.36–0.80)	0.002
Optimal Model	0.64 (0.45–0.91)^†^	0.01	0.64 (0.46–0.89)^‡^	0.008	0.56 (0.39–0.82)^§^	0.002
PS matched cohort	0.47 (0.32–0.70)	< 0.001	0.55 (0.37–0.80)	0.002	0.51 (0.34–0.78)	0.002
PS matched cohort, double robust approach^#^	0.44 (0.30–0.66)	< 0.001	0.54 (0.37–0.80)	0.002	0.51 (0.33–0.77)	0.001
Diabetes mellitus patients						
Unadjusted	0.45 (0.29–0.71)	< 0.001	0.58 (0.38–0.89)	0.01	0.44 (0.28–0.70)	< 0.001
Fully adjusted**	0.35 (0.21–0.61)	< 0.001	0.56 (0.34–0.90)	0.02	0.39 (0.23–0.65)	< 0.001
Optimal Model	0.37 (0.23–0.62)^††^	< 0.001	0.58 (0.37–0.90)^‡‡^	0.02	0.44 (0.27–0.72)^§§^	< 0.001
PS matched- Diabetes	0.39 (0.23–0.66)	< 0.001	0.58 (0.35–0.96)	0.03	0.44 (0.26–0.75)	0.003
Non-diabetes mellitus patients						
Unadjusted	1.21 (0.77–1.91)	0.42	0.72 (0.45–1.15)	0.17	0.81 (0.47–1.39)	0.45
Fully adjusted**	1.21 (0.67–2.17)	0.52	0.71 (0.41–1.25)	0.23	0.92 (0.49–1.74)	0.79
PS matched- non-Diabetes	0.95 (0.52–1.75)	0.88	0.61 (0.33–1.14)	0.12	0.82 (0.40–1.68)	0.58
Cohort of patients with history of antecedent statin use						
Unadjusted	0.56 (0.37–0.85)	0.006	0.48 (0.33–0.72)	< 0.001	0.44 (0.29–0.68)	< 0.001
Fully adjusted*	0.46 (0.28–0.74)	0.002	0.46 (0.30–0.72)	< 0.001	0.40 (0.25–0.65)	< 0.001

*Adjusted for age, sex, race, BMI, insurance, days to presentation, CURB-65 and comorbidities which include preexisting lung diseases, smoking, hypertension, coronary artery disease, Diabetes Mellitus, chronic kidney disease, ESRD on dialysis, congestive heart failure, any cancer, chronic liver disease, hyperlipidemia and history of previous stroke

^†^Variables in the optimal model- age, BMI, CURB-65 and inpatient statin use

^‡^Variables in the optimal model- age, CURB-65, Diabetes Mellitus, and inpatient statin use

^§^Variables in the optimal model- BMI, CURB-65, preexisting lung diseases, Diabetes Mellitus and inpatient statin use

^#^Adjusted for HTN

**Adjusted for age, sex, race, BMI, insurance, days to presentation, CURB-65 and comorbidities which include preexisting lung diseases, smoking, hypertension, coronary artery disease, chronic kidney disease, ESRD on dialysis, congestive heart failure, any cancer, chronic liver disease, hyperlipidemia and history of previous stroke

^††^Variables in the optimal model- age, CURB-65, preexisting lung diseases, congestive heart failure, chronic liver disease, chronic kidney disease and inpatient statin use

^‡‡^Variables in the optimal model- BMI, CURB-65 and inpatient statin use

^§§^Variables in the optimal model- BMI, CURB-65 and inpatient statin use

Similar results demonstrating significant associations between inpatient statin use and reduction in severe disease outcomes were seen in the PSM cohort. In the PSM cohort, inpatient statin use was found to be associated with reduced mortality (OR, 0.47; 95% CI, 0.32–0.70; p < 0.001), decreased need for ICU admission (OR, 0.55; 95% CI, 0.37–0.80; p = 0.002), and a lower need for mechanical ventilation (OR, 0.51; 95% CI, 0.34–0.78; p = 0.002). These results were further verified using the double robust approach to account for the difference in the distribution of patients with hypertension among the two groups (statin vs non-statin), details of which have been provided in Table 3.

### Baseline characteristics and clinical course of the patients with DM

In this study cohort, 413 patients had a documented history of DM. Median age of the patients with DM was 66 years (IQR-58–74 years), and median BMI was 29.8 kg/m^2^ (IQR- 25.7–36.15 kg/m^2^). Almost 91.5% of the patients with DM also had documented history of hypertension (n = 378). Among the COVID-19 patients with DM, 32.9% of the patients received inpatient statins (n = 136). Patients in the statins group had a higher burden of comorbidities such as CAD, hypertension, and hyperlipidemia. Baseline characteristics of the COVID-19 patients with DM have been detailed in Table 4. During the course of hospitalization, more than a third of patients with DM died (n = 152, 36.8%), about 35.4% of the patients with DM needed mechanical ventilation (n = 146), while 43.3% of the patients (n = 179) needed ICU. The details of the clinical course have been summarized in Table 2.

**Table 4:** Baseline characteristic of patients with Diabetes Mellitus

		Unmatched			Propensity score matched		
Characteristic	Diabetic Cohort (n = 413)	Inpatient Statin (n = 136)	Non Statin (n = 277)	p-value	Inpatient Statin (n = 129)	Non Statin (n = 129)	p-value
Age, (years) n (%)							
Median (IQR)	66 (58–74)	66 (59.25–75)	66 (57–73)	0.48	66 (59–75)	68 (59.5–76)	0.36
18–30	3 (0.7)	0	3 (1.1)		0	1 (0.8)	
31–45	27 (6.5)	4 (2.9)	23 (8.3)		3 (2.3)	11 (8.5)	
46–64	144 (34.9)	49 (36)	95 (34.3)		47 (36.4)	36 (27.9)	
≥ 65	239 (57.9)	83 (61)	156 (56.3)		79 (61.2)	81 (62.8)	
Sex, n (%)							
Male	217 (52.5)	68 (50)	149 (53.8)	0.47	64 (49.6)	63 (48.8)	0.9
Female	196 (47.5)	68 (50)	128 (46.2)		65 (50.4)	66 (51.2)	
Race, n (%)							
Blacks	314 (76)	101 (74.3)	213 (76.9)	0.56	97 (75.2)	97 (75.2)	1
Other races	99 (24)	35 (25.7)	64 (23.1)		32 (24.8)	32 (24.8)	
BMI							
Median (IQR)	29.8 (25.7–36.15)	29.95 (25.13–35.35)	29.8 (26.05–36.55)	0.65	30.3 (25.25–35.7)	30 (26.15–35.75)	0.79
< 18.5 (underweight)	8 (1.9)	3 (2.2)	5 (1.8)		2 (1.6)	3 (2.3)	
18.5–24.9 (normal)	82 (19.9)	29 (21.3)	53 (19.1)		28 (21.7)	20 (15.5)	
25–29.9 (overweight)	118 (28.6)	36 (26.5)	82 (29.6)		33 (25.6)	40 (31)	
≥ 30 (obese)	205 (49.6)	68 (50)	137 (49.5)		66 (51.2)	66 (51.2)	
Comorbidities, n (%)							
Preexisting lung diseases	132 (32)	46 (33.8)	86 (31)	0.57	45 (34.9)	40 (31)	0.51
Coronary artery disease	142 (34.4)	60 (44.1)	82 (29.6)	0.004	58 (45)	49 (38)	0.26
Hypertension	378 (91.5)	130 (95.6)	248 (89.5)	0.04	123 (95.3)	117 (90.7)	0.14
Hyperlipidemia	160 (38.7)	71 (52.2)	89 (32.1)	< 0.001	65 (50.4)	62 (48.1)	0.71
Congestive Heart Failure	76 (18.4)	31 (22.8)	45 (16.2)	0.11	29 (22.5)	27 (20.9)	0.76
Stroke	45 (10.9)	20 (14.7)	25 (9)	0.08	18 (14)	17 (13.2)	0.86
Cancer	35 (8.5)	11 (8.1)	24 (8.7)	0.84	11 (8.5)	13 (10.1)	0.67
Chronic liver disease	13 (3.1)	0	13 (4.7)	0.01	0	0	
Chronic kidney disease	65 (15.7)	24 (17.6)	41 (14.8)	0.46	24 (18.6)	22 (17.1)	0.75
ESRD on dialysis	53 (12.8)	18 (13.2)	35 (12.6)	0.86	18 (14)	14 (10.9)	0.45
Smoking	176 (42.6)	62 (45.6)	114 (41.2)	0.39	59 (45.7)	61 (47.3)	0.8
Insurance							
Uninsured	7 (1.7)	3 (2.2)	4 (1.4)	0.5	3 (2.3)	3 (2.3)	0.77
Medicaid	87 (21.1)	23 (16.9)	64 (23.1)		23 (17.8)	27 (20.9)	
Medicare	296 (71.7)	102 (75)	194 (70)		96 (74.4)	95 (73.6)	
Private	23 (5.6)	8 (5.9)	15 (5.4)		7 (5.4)	4 (3.1)	
Days from symptom onset to hospital presentation							
0–5	289 (70)	95 (69.9)	194 (70)	0.26	92 (71.3)	96 (74.4)	0.36
6–10	72 (17.4)	28 (20.6)	44 (15.9)		28 (21.7)	20 (15.5)	
11–15	25 (6.1)	5 93.7)	20 (7.2)		5 (3.9)	10 (7.8)	
16 +	8 (1.9)	4 (2.9)	4 (1.4)		4 (3.1)	3 (2.3)	
CURB-65 at presentation							
0	77 (18.6)	18 (13.2)	59 (21.3)	0.21	17 (13.2)	18 (14)	0.8
1	123 (29.8)	38 (27.9)	85 (30.7)		37 (28.7)	38 (29.5)	
2	145 (35.1)	58 (42.6)	87 (31.4)		56 (43.4)	48 (37.2)	
3	53 (12.8)	18 (13.2)	35 (12.6)		16 (12.4)	21 (16.3)	
4	8 (1.9)	3 (2.2)	5 (1.8)		3 (2.3)	3 (2.3)	
5	5 (1.2)	1 (0.7)	4 (1.4)		0	1 (0.8)	

In the PSM cohort of patients with DM, a 1:1 balanced cohort of 258 patients was obtained (129 patients each in the statin and non-statin group). The distribution of all the baseline characteristics was similar across the two groups in the PSM cohort of the patients with DM, the details of which have been outlined in Table 4.

### Inpatient statins and clinical outcomes in the patients with DM

In the unadjusted analyses among the patients with DM, inpatient statins use was associated with significant reduction with mortality (OR, 0.45; 95% CI, 0.29–0.71; p < 0.001), decreased need for ICU admission (OR, 0.58; 95% CI, 0.38–0.89; p = 0.01) and a reduced need for mechanical ventilation (OR, 0.44; 95% CI, 0.28–0.70; p < 0.001) (Fig. 2B). Fully adjusted regression models also yielded similar results. Diabetic patients who received inpatient statins had significantly reduced mortality (OR, 0.35; 95% CI, 0.21–0.61; p < 0.001), reduced need for ICU admission (OR, 0.56; 95% CI, 0.34–0.90; p = 0.02) as well as a decreased need for mechanical ventilation (OR, 0.39; 95% CI, 0.23–0.65; p < 0.001). These results were further verified using the optimal model obtained by Forward Wald step wise regression, the details of which have been mentioned in Table 3. In the PSM cohort of the patients with DM, inpatient statin use was found to be associated with reduced mortality (OR, 0.39; 95% CI, 0.23–0.66; p < 0.001), reduced need for ICU admission (OR, 0.58; 95% CI, 0.35–0.96; p = 0.03), and a decreased requirement for mechanical ventilation (OR, 0.44; 95% CI, 0.26–0.75; p = 0.003).

### Baseline characteristics and clinical course of the patients without DM

In the study cohort, 509 patients hospitalized with COVID-19 had no documented history of DM, out of which 22.4% of the patients received inpatient statins (n = 114). The baseline characteristics of the two groups (statin vs non-statin) among the patients without DM are shown in Table 5. During the course of hospitalization, 27.7% of the COVID-19 patients without DM (n = 141) died, 20% of the patients (n = 102) needed mechanical ventilation, and 30.6% of the patients (n = 156) needed ICU admission. The patients without DM had a less severe clinical course compared to the patients with DM. The details of analysis comparing the clinical outcomes in patients with DM to those without DM have been summarized in Additional file 1: Table S4. In PSM cohort of patients without DM, a 1:1 balanced cohort of 188 patients (94 patients each in statin and non-statin group) was obtained. The distribution of all the baseline characteristics was noted to be similar in this PSM cohort as outlined in Table 5.

**Table 5:** Baseline characteristics of patients with Non-Diabetes Mellitus

		Unmatched			Propensity score matched		
Characteristic	Cohort (n = 509)	Inpatient Statin (n = 114)	Non Statin (n = 395)	p-value	Inpatient Statin (n = 94)	Non Statin (n = 94)	p-value
Age, (years) n (%)							
Median (IQR)	65 (52–73)	67 (57.75–77)	64 (49–73)	0.006	67 (57–75.5)	67.5 (59.75–76)	0.75
18–30	23 (4.5)	0	23 (5.8)		0	2 (2.1)	
31–45	54 (10.6)	6 (5.3)	48 (12.2)		3 (3.2)	10 (10.6)	
46–64	176 (34.6)	42 (36.8)	134 (33.9)		36 (38.3)	26 (27.7)	
≥ 65	256 (50.3)	66 (57.9)	190 (48.1)		55 (58.5)	56 (59.6)	
Sex, n (%)							
Male	273 (53.6)	71 (62.3)	202 (51.1)	0.04	55 (58.5)	57 (60.6)	0.77
Female	236 (46.4)	43 (37.7)	193 (48.9)		39 (41.5)	37 (39.4)	
Race, n (%)							
Blacks	374 (73.5)	78 (68.4)	296 (74.9)	0.17	68 (72.3)	72 (76.6)	0.5
Other races	135 (26.5)	36 (31.6)	99 (25.1)		26 (27.7)	22 (23.4)	
BMI							
Median (IQR)	28.8 (24.9–35.8)	28 (23.98–34.5)	29.2 (25.4–36.43)	0.07	29.3 (24.63–34.8)	26.95 (24.33–32.7)	0.4
< 18.5 (underweight)	20 (3.9)	5 (4.4)	15 (3.8)		4 (4.3)	5 (5.3)	
18.5–24.9 (normal)	113 (22.2)	36 (31.6)	77 (19.5)		24 (25.5)	23 (24.5)	
25–29.9 (overweight)	148 (29.1)	25 (21.9)	123 (31.1)		22 (23.4)	31 (33)	
≥ 30 (obese)	227 (44.6)	48 (42.1)	179 (45.3)		44 (46.8)	35 (37.2)	
Comorbidities, n (%)							
Preexisting lung diseases	172 (33.8)	41 (36)	131 (33.2)	0.58	36 (38.3)	31 (33)	0.45
Coronary artery disease	72 (14.1)	34 (29.8)	38 (9.6)	< 0.001	26 (27.7)	22 (23.4)	0.5
Hypertension	378 (74.3)	103 (90.4)	275 (69.6)	< 0.001	84 (89.4)	76 (80.9)	0.1
Hyperlipidemia	120 (23.6)	56 (49.1)	64 (16.2)	< 0.001	38 (40.4)	37 (39.4)	0.88
Congestive Heart Failure	59 (11.6)	25 (21.9)	34 (8.6)	< 0.001	20 (21.3)	21 (22.3)	0.86
Stroke	55 (10.8)	28 (24.6)	27 (6.8)	< 0.001	18 (19.1)	20 921.3)	0.72
Cancer	53 (10.4)	7 (6.1)	46 (11.6)	0.09	7 (7.4)	4 (4.3)	0.35
Chronic liver disease	22 (4.3)	3 (2.6)	19 (4.8)	0.31	3 (3.2)	5 (5.3)	0.47
Chronic kidney disease	45 (8.8)	17 (14.9)	28 (7.1)	0.01	12 (12.8)	13 (13.8)	0.83
ESRD on dialysis	45 (8.8)	13 (11.4)	32 (8.1)	0.27	12 (12.8)	10 (10.6)	0.65
Smoking	211 (41.5)	55 (48.2)	156 (39.5)	0.1	43 (45.7)	48 (51.1)	0.47
Insurance							
Uninsured	2 (0.4)	0	2 (0.5)	0.08	0	1 (1.1)	0.44
Medicaid	155 (30.5)	29 (25.4)	126 (31.9)		21 (22.3)	23 (24.5)	
Medicare	299 (58.7)	78 (68.4)	221 (55.9)		66 (70.2)	67 (71.3)	
Private	53 (10.4)	7 (6.1)	46 (11.6)		7 (7.4)	3 (3.2)	
Days from symptom onset to hospital presentation							
0–5	360 (70.7)	90 (78.9)	270 (68.4)	0.06	77 (81.9)	66 (70.2)	0.25
6–10	88 (17.3)	11 (9.6)	77 (19.5)		9 (9.6)	18 (19.1)	
11–15	30 (5.9)	5 (4.4)	25 (6.3)		5 (5.3)	6 (6.4)	
16 +	10 (2)	3 (2.6)	7 (1.8)		3 (3.2)	4 (4.3)	
CURB-65 at presentation							
0	145 (28.5)	18 (15.8)	127 (32.2)	0.004	14 (14.9)	21 (22.3)	0.17
1	157 (30.8)	43 (37.7)	114 (28.9)		36 (38.3)	27 (28.7)	
2	129 (25.3)	30 (26.3)	99 (25.1)		27 (28.7)	30 (31.9)	
3	57 (11.2)	20 (17.5)	37 (9.4)		15 (16)	11 (11.7)	
4	12 (2.4)	1 (0.9)	11 (2.8)		0	4 (4.3)	
5	7 (1.4)	2 (1.8)	5 (1.3)		2 (2.1)	1 (1.1)	

### Inpatient statins and clinical outcomes in patients without DM

Unlike the COVID-19 patients with DM, the COVID-19 patients without DM did not have any significant association between inpatient statin use and the clinical outcomes explored by this study (Fig. 2C). We did not find any significant association between inpatient statins and mortality, the need for ICU admission or the need for mechanical ventilation among the COVID-19 patients without DM in the unadjusted and fully adjusted models. These results were further verified in the PSM cohort of patients without DM and have been summarized in Table 3.

### Inpatient statins in patients using statins as home medications

This study also explored the association between inpatient statins and clinical outcomes in the subset of patients who were antecedent statin users, i.e. the patients using statins as home medication. A total of 438 (47.5%) patients were using statins as home medication at the time of presentation to the hospital. Out of these patients, statin therapy was continued in the inpatient setting for 217 (49.5%) patients (Table 6). Upon the review of medical records, a documented reason for discontinuation of statins in the inpatient setting for antecedent statin users (rhabdomyolysis, transaminitis, hospital stay less than 48 h, patient’s inability to tolerate oral medications or myalgias) could only be determined for less than 50% of the patients. Antecedent statin users who were continued on statin therapy in the hospital had reduced mortality, decreased need for ICU admission, and decreased need for mechanical ventilation compared to the antecedent statin users who did not receive statins in the hospital. The results have been summarized in Table 3.

**Table 6:** Description of antecedent statin use, if continued during admission and reason for discontinuation

Characteristic	Total cohort
Home statins documented	438 (47.5)
Continued inpatient statins	217 (49.5)
New inpatient statins	33 (13.2)
Reason for discontinued home statins	
Rhabdomyolysis	20 (9.2)
Transaminitis	33 (15.2)
Cannot tolerate oral medications	26 (12)
Admission < 48 h	18 (8.3)
Myalgias	3 (1.4)
Cannot be determined	117 (53.9)
Reason for new inpatient statins	
NSTEMI	12 (36.4)
Acute stroke	4 (12.1)
New Heart Failure	1 (3)
New diabetes	3 (9.1)
Cannot be determined	13 (39.4)

### Intensity-response relationship of statins with clinical outcomes

Further exploratory analyses revealed a significant intensity-response relationship of inpatient statins for reduced mortality and need for mechanical ventilation among the hospitalized COVID-19 patients. In the PSM total cohort as well PSM-DM cohort, compared to patients who did not receive any inpatient statins, patients who received a low intensity did not have any significant reduction in mortality, but the patients who received moderate and high intensity of statins had significantly reduced mortality. However, only 8% of the patients received low intensity statins (n = 20), hence the analysis of the patients who received low intensity statins was statistically underpowered. The details of these analyses for both the total cohort and DM cohort have been summarized in Table 7.

**Table 7:** Association between inpatient statin intensity and severe disease outcomes- Mortality, Mechanical ventilation and ICU admission

Characteristic	Mortality		ICU Admission		Mechanical ventilation	
	OR (95% CI)	p-value	OR (95% CI)	p-value	OR (95% CI)	p-value
Total cohort*						
No statin	Ref		Ref		Ref	
Low	0.35 (0.11–1.07)	0.07	0.58 (0.21–1.63)	0.3	0.71 (0.24–2.09)	0.53
Moderate	0.61 (0.35–1.06)	0.08	0.65 (0.39–1.09)	0.11	0.49 (0.27–0.90)	0.02
High	0.70 (0.44–1.13)	0.14	0.62 (0.40–0.96)	0.03	0.59 (0.36–0.95)	0.03
PSM- Total cohort						
No statin	Ref		Ref		Ref	
Low	0.31 90.09–1.13)	0.08	0.29 (0.08–1.04)	0.06	0.45 (0.12–1.61)	0.22
Moderate	0.40 (0.23–0.72)	0.002	0.54 (0.31–0.92)	0.02	0.43 (0.23–0.80)	0.007
High	0.54 (0.34–0.86)	0.009	0.59 (0.38–0.93)	0.02	0.58 (0.36–0.95)	0.03
Diabetes Mellitus patients^†^						
No statin	Ref		Ref		Ref	
Low	0.14 (0.03–0.76)	0.02	0.28 (0.07–1.19)	0.09	0.35 (0.08–1.51)	0.16
Moderate	0.47 (0.22–0.98)	0.04	0.51 (0.25–1.03)	0.06	0.53 (0.26–1.09)	0.09
High	0.36 (0.18–0.70)	0.003	0.71 (0.40–1.29)	0.26	0.35 (0.18–0.69)	0.003
PSM- Diabetes Mellitus						
No statin	Ref		Ref		Ref	
Low	0.26 (0.06–1.27)	0.1	0.41 (0.10–1.60)	0.2	0.31 (0.06–1.49)	0.14
Moderate	0.45 (0.22–0.94)	0.03	0.51 (0.25–1.02)	0.06	0.53 (0.26–1.10)	0.09
High	0.37 (0.20–0.71)	0.003	0.66 (0.37–1.20)	0.17	0.40 (0.21–0.78)	0.007

*Adjusted for age, sex, race, BMI, insurance, days to presentation, CURB-65 and comorbidities which include preexisting lung diseases, smoking, hypertension, coronary artery disease, diabetes mellitus, chronic kidney disease, ESRD on dialysis, congestive heart failure, any cancer, chronic liver disease, hyperlipidemia and history of previous stroke

^†^Adjusted for age, sex, race, BMI, insurance, days to presentation, CURB-65 and comorbidities which include preexisting lung diseases, smoking, hypertension, coronary artery disease, chronic kidney disease, ESRD on dialysis, congestive heart failure, any cancer, chronic liver disease, hyperlipidemia and history of previous stroke

## Discussion

In this retrospective cohort study of the majority Black population, inpatient statin use was found to be associated with decreased all-cause mortality, reduced need for ICU admission, and decreased requirement for mechanical ventilation among the hospitalized COVID-19 patients. This study accounted for most of the known confounders such as demographics, BMI, smoking status, days from symptom onset to presentation, CURB-65 score at presentation, and 11 most prevalent comorbidities. Similar results were obtained using multivariable regression models as well after propensity score matching to minimize the selection bias and the effect of known confounders. Interestingly, the presence of DM was an important factor affecting this association between inpatient statins and clinical outcomes. Patients with DM who received inpatient statins had close to 60% reduction in all-cause mortality, about 40% reduction in the need for ICU admission and about 55% decrease in the need for mechanical ventilation compared to the patients with DM who did not receive inpatient statins, in the fully adjusted and PSM models of this study. On the contrary, none of the clinical outcomes explored by this study had any significant association with inpatient statin use in the COVID-19 patients without DM.

A couple of large multi-hospital cohort studies have demonstrated close to 40% reduction in all-cause mortality among COVID-19 patients who received inpatient statins, similar to what has been reported in our study [28, 29]. Saeed et al. [24] reported 49% reduction in mortality with inpatient statin use in COVID-19 patients with DM compared to about 60% reduction noted in our study. However, their baseline population consisted of only 37% Black patients compared to 75% Black population in our study cohort. These results assume greater significance given the worse clinical outcomes and increased hospitalization due to COVID-19 among the underserved and Black patient population [30–33].

DM is one of the most common chronic conditions identified among patients with COVID-19 and associated with worse clinical outcomes [34–37]. Impaired adaptive immunity and a late hyperinflammatory response seen in DM [38], has been hypothesized as a possible mechanism for increased inflammation in COVID-19 patients with DM [39, 40]. Plausible explanations for the possible benefits of statins seen in this patient population could include anti-inflammatory and immunomodulating properties of statins, including T cell signaling, modulation of cytokine production [16, 17], protection against vascular injury and endothelial dysfunction [13–15], inhibition of MYD88 gene and thereby activation of NF-kb pathway [41, 42], and pleiotropic effect on NLRP3 inflammasome activation and cytokine release [43, 44].

It has been well established in the literature on COVID-19 that patients with DM have worse clinical outcomes, similar to what has been seen in this study. Recent observational studies have demonstrated a significant association between antecedent statin use and reduced mortality in the patients presenting to the hospital with COVID-19 [19–23]. However, to our knowledge, the only other study exploring the association between clinical outcomes and continuation of statins in the inpatient setting among the hospitalized COVID-19 patients who were using statins as home medication is by Masana et al. [45] from Spain. In their study cohort, home statins were continued in 57.5% of the patients during hospitalization, and these patients had reduced mortality compared to patients for whom statins were discontinued, although this difference was not statistically significant. However, patients in their study had less severe burden of comorbidities compared to our cohort and the race information was missing. In our study, we noted reduced mortality, decreased need for ICU admission, and decreased need for mechanical ventilation in the patients who were continued on statins in the hospital compared to those patients who were discontinued on statins in the inpatient setting, among the antecedent statin users. These findings indicate that continuation of statin therapy seems to be safe in hospitalized COVID-19 patients who were using statins as home medications, unless they present with one of the contraindications for statin administration.

Although this methodologically strong study reports some clinically relevant findings, some limitations need to be acknowledged. This is a retrospective observational study, and the results can only imply association and not causation. Due to the nature of the study, there is a possibility of selection bias and residual confounding due to unknown confounders. Also, this study could not look at the concomitant therapies such as the use of angiotensin inhibitors, aspirin etc. Most of the patients who received inpatient statins were also antecedent statin users at home, thereby this study cannot delineate between the long term and immediate benefit of statins. Also, the information about the median time from admission to the beginning of inpatient statin therapy was not available. Additionally, majority of patients in this study cohort were Blacks which may limit the generalization of the results. Among the antecedent statin users, the compliance to statin home medications could not be assessed. This study did not look at the chronicity of DM, complications resulting from DM, or how well it was controlled in the patients of the study cohort. Randomized control trials are needed to provide the best evidence regarding the efficacy of statins in reducing mortality in the patients hospitalized with COVID-19, especially those with preexisting DM. Further studies on statins and DM should consider exploring if the chronicity of the disease, glycated hemoglobin levels, presence of microvascular and macrovascular complications from DM, etc. have an effect on the association between statins and reduced disease severity of COVID-19.

## Conclusion

This observational study reports that inpatient statin use was associated with reduced mortality, decreased need for ICU admission and a lower need for mechanical ventilation among hospitalized COVID-19 patients with preexisting DM. In addition, this study identifies reduction in the above severe disease outcomes among antecedent statin users who were continued on statins in the hospital, compared to those whose statins were discontinued in the hospital. These results indicate that statin therapy appears to be safe in patients hospitalized with COVID-19, especially those with DM, and warrant the pursuit for randomized control trials to ascertain the benefits of statins.

## Supplementary Information

**Additional file 1: Table S1.** Definitions of baseline characteristics, comorbidities and treatment. **Table S2.** Description of treatments received during hospitalization. **Table S3.** Description of intensity and type of inpatient statins. **Table S4.** Association between Diabetes Mellitus and severe disease outcomes- Mortality, Mechanical ventilation and ICU admission.

## Data Availability

The de-identified data that support the findings of this study can be available from the corresponding author upon reasonable request and appropriate permission from the institutional IRB.

## Abbreviations

COVID-19: Coronavirus disease
SARS-CoV-2: Severe acute respiratory syndrome coronavirus-2
DM: Diabetes Mellitus
US: United States
ICU: Intensive care unit
DMC: Detroit Medical Center
ED: Emergency department
ECMO: Extracorporeal membrane oxygenation
EMR: Electronic medical records
CAD: Coronary artery disease
CHF: Congestive heart failure
CKD: Chronic kidney disease
ESRD: End stage renal disease
OR: Odds ratio
CI: Confidence interval
SD: Standard Deviation
BUN: Blood urea nitrogen
PSM: Propensity score matched
TC: Total cohort
