# Supplementary material for: Statins and clinical outcomes in hospitalized COVID-19 patients with and without Diabetes Mellitus: a retrospective cohort study with propensity score matching
Source: Cardiovasc Diabetol. 2021 Jul 10;20:140. doi: 10.1186/s12933-021-01336-0 (PMC8272452; doi:10.1186/s12933-021-01336-0)
Supplement: Supplementary file 1 — Additional file 1: Table S1. Definitions of baseline characteristics, comorbidities and treatment. Table S2. Description of treatments received during hospitalization. Table S3. Description of intensity and type of inpatient statins. Table S4. Association between Diabetes Mellitus and severe disease outcomes- Mortality, Mechanical ventilation and ICU admission. [file 12933_2021_1336_MOESM1_ESM.docx]

**Statins and clinical outcomes in hospitalized COVID-19 patients with and without Diabetes Mellitus: a retrospective cohort study with propensity score matching**

**Supplementary tables**

Supplementary table 1- Definitions of baseline characteristics, comorbidities and treatment

Supplementary table 2- Description of treatments received during hospitalization

Supplementary Table 3- Description of intensity and type of inpatient statins

Supplementary table 4: Association between Diabetes Mellitus and severe disease outcomes- Mortality, Mechanical ventilation and ICU admission

| **Supplementary table 1- Definitions of baseline characteristics, comorbidities and treatments** | |
| --- | --- |
| **Baseline Characteristics** |  |
| Smoking status | Per chart review, current or former smoker |
| Insurance status | Per chart review, Medicaid, Medicare, Private or uninsured |
| **Comorbidities** |  |
| Diabetes Mellitus | Per chart review, Insulin and Non-insulin dependent |
| Hypertension | Per chart review |
| Coronary artery disease | Per chart review, any history of angina, myocardial infarction, stent placement or coronary artery bypass graft surgery |
| Congestive heart failure | Per chart review, heart failure with preserved versus reduced ejection fraction |
| Hyperlipidemia | Per chart review |
| Chronic kidney disease | Per chart review, baseline eGFR<60 (excludes any patients on current hemodialysis or peritoneal dialysis) |
| Preexisting lung disease | Per chart review, COPD, Asthma, Obstructive sleep apnea, Sarcoidosis, Interstitial; lung disease, pulmonary hypertension, any lung cancer, history of pulmonary embolism, tuberculosis |
| End stage renal disease on dialysis | Per chart review, patients on hemodialysis or peritoneal dialysis |
| Chronic liver disease | Per chart review, Cirrhosis, Alcohol related liver disease, nonalcoholic fatty liver disease, autoimmune hepatitis, Hepatitis B or C, primary biliary cirrhosis. |
| Cancer | Per chart review (other than non-melanoma skin cancer), cancer of the breast, colorectal, prostate, gastric, pancreatic, melanoma, brain, ovarian, blood or other |
| Stroke | Per chart review; ischemic or hemorrhagic |
| **Treatment** |  |
| Antibiotics | Oral or intravenous antibiotics for pneumonia during admission |
| Remdesivir | Per chart review, Criteria- laboratory confirmed SARS-CoV-2, respiratory symptoms onset ≤10 days, SpO2 ≤94% on room air and requiring supplemental oxygen, radiographic evidence of pulmonary infiltrates on Chest Xray or CT scan |
| Anticoagulation | Therapeutic anticoagulation (continued home anticoagulation therapy if indicated or), Criteria - New deep vein thrombosis, pulmonary embolism or D-dimer ≥ 3mg/L at admission |
| Corticosteroids | Per chart review, dexamethasone, methylprednisolone, prednisone or hydrocortisone. Criteria- SpO2 ≤94% on room air, new supplemental oxygen requirement or mechanical ventilation |

| **Supplementary Table 2- Description of treatments received during hospitalization** | | | | |
| --- | --- | --- | --- | --- |
| **Characteristic** | **Cohort (n= 922)** | **Statin (n= 250)** | **Non-Statin (n=672)** | **p-value** |
| Corticosteroids | 310 (33.6) | 89 (35.6) | 221 (32.9) | 0.44 |
| Remdesivir | 8 (0.9) | 3 (1.2) | 5 (0.7) | 0.51 |
| Anticoagulation | 185 (20.1) | 51 (20.4) | 134 (19.9) | 0.88 |
| Antibiotics | 731 (79.3) | 196 (78.4) | 535 (79.6) | 0.69 |

| **Supplementary Table 3- Description of intensity and type of inpatient statins** | | | | | | |
| --- | --- | --- | --- | --- | --- | --- |
| **Intensity of Statins** | **Total Cohort** | **PSM Total Cohort** | **Diabetes Mellitus** | **PSM Cohort Diabetes Mellitus** | **Non-Diabetes Mellitus** | **PSM Non-Diabetes Mellitus** |
| Low | 20 (8) | 16 (7) | 12 (8.8) | 12 (9.3) | 8 (7) | 5 (5.3) |
| Moderate | 89 (35.6) | 83 (36.2) | 49 (36) | 47 (36.4) | 40 (35.1) | 34 (36.2) |
| High | 141 (56.4) | 130 (56.8) | 75 (55.1) | 70 (54.3) | 66 (57.9) | 55 (58.5) |
| **Type of inpatient statins** | | | | | | |
| Atorvastatin | 205 (82) | 191 (83.4) | 115 (84.6) | 107 (82.9) | 91 (79.8) | 76 (80.9) |
| Pravastatin | 23 (9.2) | 20 (8.7) | 11 (8.1) | 12 (9.3) | 11 (9.6) | 9 (9.6) |
| Rosuvastatin | 16 (6.4) | 13 (5.7) | 6 (4.4) | 6 (4.7) | 10 (8.8) | 8 (8.5) |
| Simvastatin | 6 (2.4) | 5 (2.2) | 4 (2.9) | 4 (3.1) | 2 (1.8) | 1 (1.1) |

| **Supplementary Table 4: Association between Diabetes Mellitus and severe disease outcomes- Mortality, Mechanical ventilation and ICU admission** | | | | | | |
| --- | --- | --- | --- | --- | --- | --- |
| **Characteristic** | **Mortality** | | **ICU Admission** | | **Mechanical ventilation** | |
|  | **OR (95% CI)** | **p-value** | **OR (95% CI)** | **p-value** | **OR (95% CI)** | **p-value** |
| Unadjusted | 1.52 (1.15-2.01) | 0.003 | 1.73 (1.32-2.27) | <0.001 | 2.18 (1.62-2.93) | <0.001 |
| Fully adjusted* | 1.43 (1.03-1.99) | 0.03 | 1.80 (1.33-2.44) | <0.001 | 2.14 (1.54-2.98) | <0.001 |
| *Adjusted for age, sex, race, BMI, insurance, days to presentation, CURB-65 and comorbidities which include preexisting lung diseases, smoking, hypertension, coronary artery disease, chronic kidney disease, ESRD on dialysis, congestive heart failure, any cancer, chronic liver disease, hyperlipidemia and history of previous stroke | | | | | | |
